# Behavioural and welfare implications of a new slipping methodology for purse seine fisheries in Norwegian waters

**DOI:** 10.1371/journal.pone.0213031

**Published:** 2019-03-11

**Authors:** Neil Anders, Mike Breen, Jostein Saltskår, Bjørn Totland, Jan Tore Øvredal, Aud Vold

**Affiliations:** 1 Fish Capture Division, Institute of Marine Research (IMR), Bergen, Norway; 2 Department of Biological Sciences, University of Bergen, Bergen, Norway; MARE – Marine and Environmental Sciences Centre, PORTUGAL

## Abstract

The release of unwanted fish from purse seines whilst still in the water is termed slipping and may lead to significant mortality following release. The objective of this study was to determine the fish welfare implications of a new slipping methodology in which fish are released via a discharge opening formed in the bunt end of the purse seine net. Video analyses of collective and individual level fish behaviour were undertaken in the Norwegian mackerel and herring purse seine fisheries, to quantitively describe slipping behaviour and to determine its driving factors. The majority of fish escaped the purse seine with the schooling structure intact as part of large groups towards the end of slipping process, increasing their speed following escape. However, there was also a tendency (24% of all escapes) to escape in a manner likely to impact negatively upon their welfare, with a breakdown in schooling structure and physical contact with the fishing gear and conspecifics. The tendency to express such welfare compromising behaviour was higher for mackerel than for herring, but was also influenced by the vessel releasing the fish, the amount of fish being slipped, how long the discharge opening had been open and the particular slipping event. These results provide important information for future science-based development of welfare friendly slipping practises.

## Introduction

Purse seining is a widespread [1], effective [2] and relatively fuel efficient [3–6] capture method for small pelagic schooling species. However, it is not without challenges. A lack of suitable monitoring technology means that skippers typically lack detailed information regarding school size and characteristics prior to setting the net [7]. This can lead to discarding when resulting catches are undesirable in some way. Discarding can be detrimental to sustainable fishery management as discarded fish may die [8,9], which can introduce uncertainty into stock assessment if not properly accounted for [10–12].

Discarding in purse seining often takes the form of slipping. Slipping is a somewhat unique case of discarding, in that the unwanted catch (or component that is unwanted) is released whilst still in the water and before being brought aboard the fishing vessel [13–15]. As such, releasing unwanted catches via slipping offers an opportunity to release fish without exposing them to the additional stressors associated with onboard catch sorting, such as air exposure [16]. Despite this, previous work has demonstrated that mortality rates for a variety of small pelagic fish species after release via slipping can be significant [17–21]. This potentially low rate of survival is related to the degree and duration of crowding in the final stages of capture [17,19,20], with mortality likely being induced via mechanisms such as hypoxia, physical injury, scale loss and exhaustion.

This potential for low survival following slipping is recognized in the management of the extensive Norwegian purse seine fisheries for Atlantic mackerel (*Scomber scombrus*). To minimize detrimental effects on survivability, legislation dictates that if mackerel are to be slipped then the net must be opened before potentially harmful levels of crowding are reached and that the opening should be of sufficient size to ensure that the fish can swim out freely [22]. It is thought that Atlantic herring (*Clupea harengus*) are more robust to the negative effects of slipping than mackerel [21] and consequently similar legislation does not exist for the Norwegian herring fishery. However, for both species, there is currently limited understanding of how the process of slipping itself impacts upon the welfare of the fish.

Despite being a contentious issue [23–27], an appreciation of fish welfare in commercial fishing has greater utility than just addressing ethical concerns. It is a useful framework by which to identify capture induced stress, which is known to affect not only product quality [28] but also subsequent survival in released catches [19]. An understanding of welfare is of particular importance here because anecdotal evidence suggests slipping may be a routine part of many purse seine fisheries [19], especially when schooling density is high (resulting in unavoidably large catch sizes which exceed quota limits or vessel capacity) or when higher prices may be obtained for certain fish species, sizes or quality [7].

It is now well established that individual behaviour and behavioural change can be useful in determining the welfare status of fish [16,25,29,30]. However, as both mackerel and herring are obligate schoolers [31] and as such spend much of their lives interacting in tight polarized groups with conspecifics, our contention is that the welfare status of such fish is best determined by examination of not only their individual behaviour, but also their collective, school-level behaviour.

In Norway, a new best practice methodology for purse seine slipping was developed in 2014 in conjunction with fishers, managers and scientists with the aim of reducing impacts upon fish slipped from purse seines. To determine the implications of this methodology on fish welfare, the aim of this study was to describe the behaviour of mackerel and herring whilst being slipped. As an understanding of the drivers of behaviour may allow it to be manipulated in the desired direction [32], a secondary objective was to determine factors influencing the observed behaviour. We were particularly interested in slipping behaviours and drivers that negatively impacted upon the animals’ welfare, to better inform future science-based regulation of slipping practices which maximize the survival potential of released catches.

## Materials and methods

### Vessels, gear and slipping methodology

**Fig 1:**
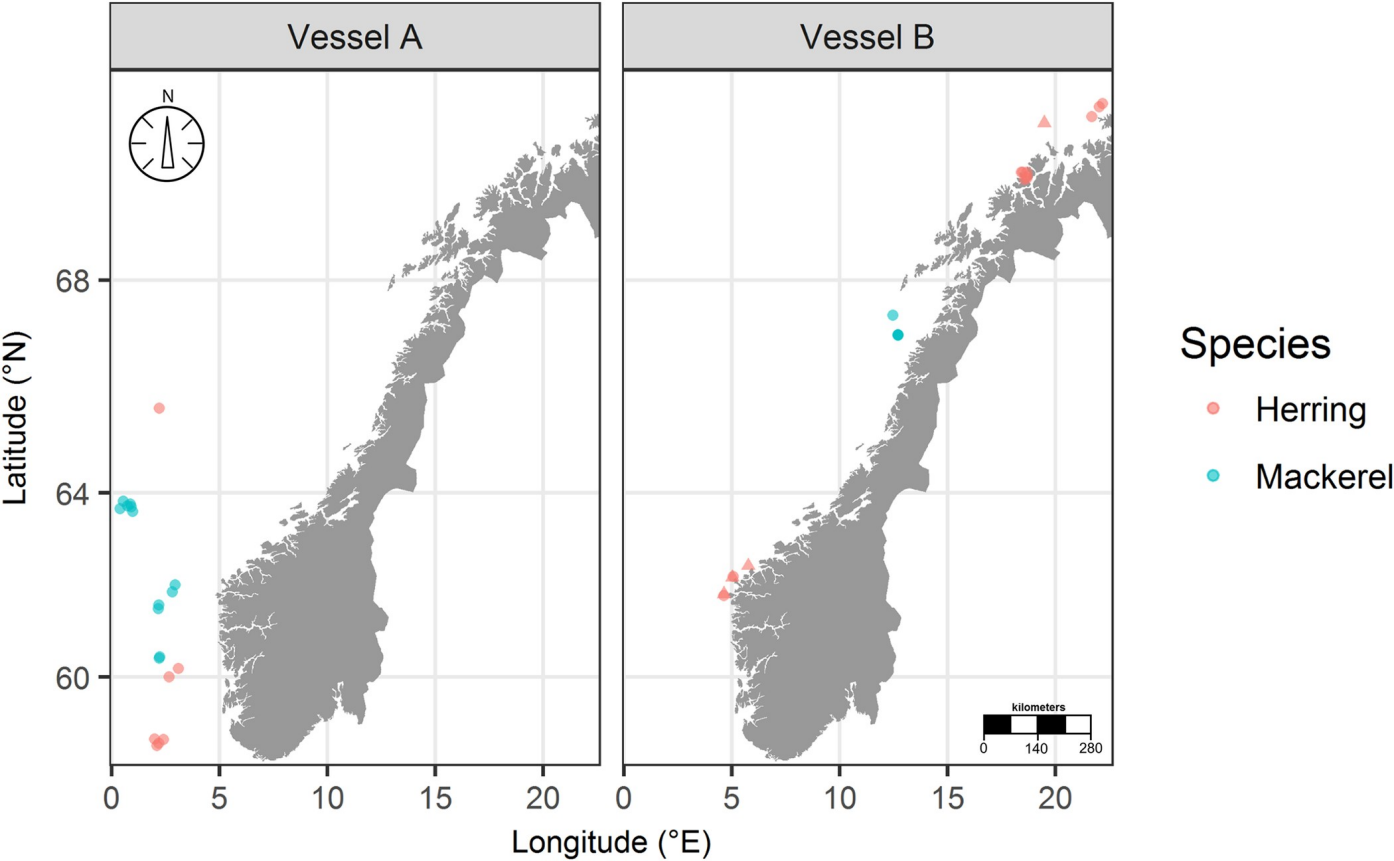
**Spatial positions of mackerel and herring slipping events for Vessel A and Vessel B.** Triangles denote slipping events not included in the analysis due to no behaviour being recorded; circles denote slipping events included in the analysis. Note that some points overlap.

Behavioural observations of mackerel and herring during slipping were collected during experimental fishing in 2015 and 2016, at coastal and offshore locations in the North and Norwegian Seas (Fig 1). Two different commercial purse seine vessels were used: an offshore vessel named here as Vessel A (LOA 64.2m) and a coastal vessel (Vessel B, LOA 36.3m). Vessel A fished with a purse seine of 746m long, with a depth of 212m, while Vessel B fished with a 571m long by 201m deep net. These dimensions represent the typical net sizes of the Norwegian purse seining fleet.

**Fig 2:**
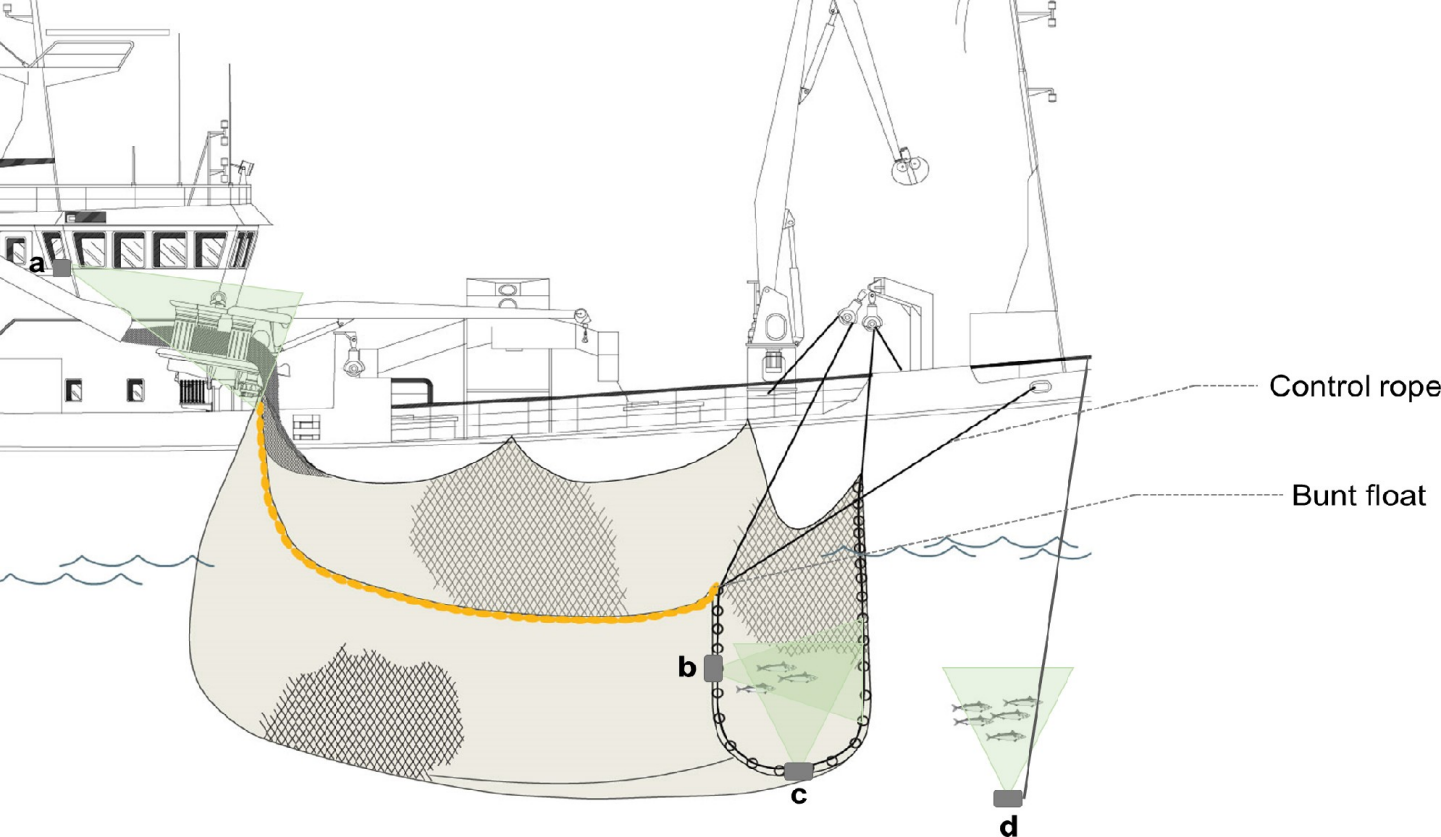
Illustration of the discharge opening and camera positioning. The discharge opening was formed in the bunt end of the purse seine to allow fish escape. The positioning of cameras (and approximate filming orientation in green) for observation of behaviour is indicated: a) bridge camera to observe net hauling and discharge opening from the surface; b) horizontally orientated discharge camera; c) vertically orientated discharge camera and d) vertically orientated drop camera. See main text for further description of camera set up and slipping methodology. Adapted from [7], with permission IMR.

Both vessels employed the “best practice” methodology guidelines for slipping, meaning that: 1) the release of fish took place through an opening (hereafter, the discharge opening) formed by the terminal end of the net bunt (Fig 2); 2) the length of the bunt end from end to end was a minimum of 18m [33]; 3) the “hang-in” ratio in the net bunt was a maximum of 25% (which is the equivalent of a minimum “hanging ratio”, E, of 0.8; see [34]) and 4) a control rope was attached to the bunt float in order to control the size of the discharge opening (Fig 2). Further explanation of purse seine terminology can be found in [2] and [7]. These criteria were intended to make the slipping procedure easier to control and to maximize the size of the discharge opening available for fish to escape through. Furthermore, when applicable, legislation regarding mackerel slipping was also followed, meaning that the discharge opening was formed before 7/8^th^ of the net length was brought onboard to avoid detrimental levels of crowding [22].

In practice, fishing followed typical commercial practices of ensonifying the school, followed by shooting, pursing and hauling of the net. The point of 7/8^th^ of the net length was marked with a white buoy, as a clearly visible indicator. Prior to this point being hauled onboard, the discharge opening was typically closed, bunched against the vessel’s side using a rope running through rings in the bunt end. To create the opening, the bunt end rope was paid out with the width and depth being adjusted as desired by the control rope attached to the bunt float. Control of width and depth allowed rapid alteration to the discharge opening in response to fish movements and behaviour as well as changes in the position and orientation of the net and vessel. Whilst the discharge opening was open, the net was hauled continuously at a steady and controlled rate. Similar to typical practice in commercial fishing, catches were either partially slipped (in which the discharge opening was closed once the desired amount of fish had been released, determined using a combination of prior experience, visual estimation and slipping time) or completely slipped (in which the opening remained open until the net was completely hauled aboard).

### Video camera positioning

Slipping events were monitored using either GoPro HERO3, HERO4 or HERO5 cameras, recording in high definition colour with a minimum of 1080p at 25fps. One camera (hereafter the “bridge camera”) was positioned on the starboard bridge wing to record the hauling of the net and the discharge opening from the surface (Fig 2). Two cameras (hereafter the “discharge cameras”) with waterproof housings were encased in Divinycell foam casings (to provide buoyancy and protection) and attached to the bunt end to record behaviour as fish escaped through the discharge opening (Fig 2). One camera was situated approximately 1m out from the midpoint of discharge opening and was orientated to film vertically upwards. The second camera was initially situated approximately 3m inwards from the midpoint of the discharge opening and oriented to film horizontally. However, the field of view was often blocked by netting in this position, so the position was changed to approximately 7m from the bunt float on subsequent deployments (data collected from both positions were used in the analysis). When light conditions were poor, discharge cameras were deployed with a red LED light (Brinyte Model: DIV01C-V, www.brinyte.com) for additional lighting. A fourth camera (hereafter the “drop camera”) was lowered into the water on a rope, approximately 5m forward from the discharge opening to record behaviour after the fish had escaped the net (Fig 2). It was attached to a frame with a wing, which stabilised the camera whilst filming by preventing the camera from spinning at the end of the rope by orientating the camera into any current. The drop camera was positioned deeper than the discharge opening and filmed vertically upwards towards the surface. The maximum number of cameras employed per slipping event was therefore four.

### Behaviour description and quantification

**Fig 3:**
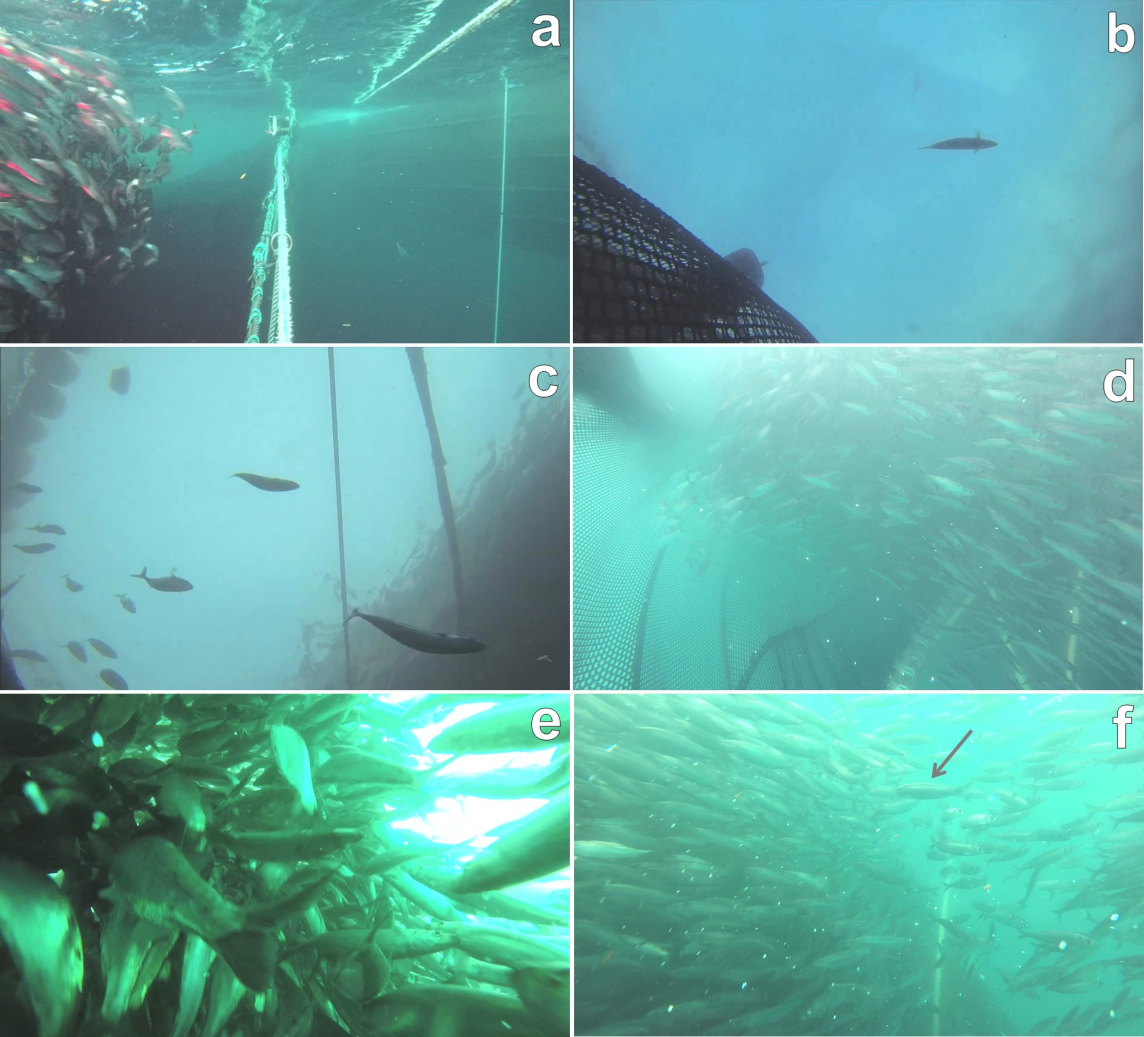
Examples of behavioural units. Behavioural units were used to classify collective fish behaviour during escape from purse seines: a) “No escape”; b) “Single fish”; c) “Small group”; d) “Orderly”; e) “Disorderly”; f) “Return”, with a fish re-entering the net indicated by the arrow. See Table 1 for full definition of behavioural units.

Video footage from the discharge cameras was synchronised with respect to time, allowing observation of slipping behaviour from two different perspectives; horizontal and vertical. Preliminary observations were then used to construct an ethogram of collective slipping behaviour, considering the size of the group escaping the net and their schooling structure (Table 1 and Fig 3). Two types of behavioural units were classified, either states (mutually exclusive, prolonged behaviours) or events (discrete, short duration behaviours [35]). Behavioural units were then assigned a hypothesized “welfare impact” (Table 1) depending on the likely impact upon individual fish. We focused on a function- and nature-based approach to welfare (in accordance with [23]), in that we defined behavioural units which were likely to cause injuries and/or were expressions of dysfunctional behaviour (equating, for example, in an obligate schooling species to a loss of schooling structure or individuals swimming alone) as having negative welfare impacts. Video examples of behavioural units and typical camera perspectives are given in the supporting information (S1–S7 Videos).

**Table 1:** Slipping behaviour ethogram. Ethogram of collective fish behaviour during escape from purse seines.

Behavioural unit	Type	Description	Hypothesised welfare impact
No escape	State	Discharge channel (or its immediate area) in field of view of cameras, but with no fish escaping the net	Uncertain–positive impacts resulting from maintenance of schooling; negative impacts resulting from continued risk of impact of capture stressors
Single fish	Event	Discharge channel (or its immediate area) in field of view of camera, with a single fish escaping the net	Moderate–loss of schooling advantages in an obligatory schooling species
Small group	Event	Discharge channel (or its immediate area) in field of view of camera, with a small group^1^ of fish (<30) escaping the net with a coordinated schooling structure	Minor–school structure remains intact, low probability of contact related injuries
Orderly	State	Discharge channel (or its immediate area) in field of view of camera, with a large group of fish (>100) escaping^3^ the net with a coordinated schooling structure	Minor–school structure remains intact, low probability of contact related injuries
Disorderly	State	Discharge channel (or its immediate area) in field of view of camera, with a large group of fish (>100) escaping^3^ the net without a coordinated schooling structure, often with collisions between fish and into gear	High–schooling structure lost in an obligatory schooling species, high probability of contact related injuries
Disagreement	State	Discharge channel (or its immediate area) in field of view, but with disorderly behaviour and orderly behaviour evident on different cameras	Minor / High–as for orderly or disorderly above.
Return	Event	Discharge channel (or its immediate area) in field of view of camera and an escape taking place, with ≥ 1 fish of the group re-entering the net	Uncertain–positive impacts resulting from joining large school inside the net; negative impacts resulting from increased risk of impact from capture stressors
Not in view	State	Either: 1) both camera views obscured, or 2) discharge channel (or its immediate area) not in field of view of either camera	NA

^1^A group was defined as >1 fish in close proximity (less than approximately 5 body lengths distance between individuals)

^2^A coordinated structure was defined as a polarized group of fish, capable of collective schooling movements

^3^The start and end of an escape for groups of >100 fish was defined as when the first and last fish passed the discharge channel threshold.

The total duration of each behavioural state and the frequency of events was then quantified over the whole duration of the slipping event, using the event logging software Observer XT 12.0 (Noldus Information Technology, www.noldus.com). Both horizontal and vertical perspectives were considered together when classifying behaviour. Only the escape behaviour of the target species of mackerel and herring were considered; behaviour of the minimal amount of bycatch (non-target species) was not included. The start of slipping was defined as the point at which the discharge opening was opened, as recorded by the bridge camera. When the whole catch was slipped, the end of slipping was defined as the point at which the bunt float reached the Triplex. For partial slipping, the end of slipping was defined as the point at which the discharge opening was closed again. It was necessary to include a “not in view” behavioural state (Table 1), as the floatation attached to the discharge cameras meant they were free to move and turn with the gear and current, resulting in times when the discharge channel was not in the field of views or the lenses was covered by netting.

To determine the effects of slipping upon behaviour at the level of individual fish, we quantified tail beat frequency (TBF) at two different stages of the slipping process; during and after escape. Changes in activity in response to stress for small pelagic species has been noted previously [36–38] and TBF is a key determinant of both swimming speed [39] and energy expenditure in fish [40–43]. TBF during escape was collected from the vertically orientated discharge camera. TBF after escape was collected from the drop camera. Random sampling of individuals within time intervals was employed. The elapsed time between the first and last appearance of escaping fish on the camera was divided into five equal time intervals. Following this, video frames from within each time interval was converted to a sequence of still images. A random starting image from within each sequence was then selected and viewed using ImageJ V1.51 software [44]. Starting images were then overlaid with a grid, allowing random selection of fish within the image by generating random grid coordinates. The TBF of five fish from within each time interval was then quantified over the duration of their appearance on subsequent images. If no fish was present at the grid coordinate, another coordinate was generated. To gather a more accurate measure of TBF, selected fish which appeared on camera for less than one second were rejected, and another random fish was selected for sampling instead. A single tail beat was defined as the movement from one extreme lateral position to the opposite extreme lateral position. Whether selected fish were escaping as part of a large group (> 100 individuals, Table 1) or not was also noted.

### Potential explanatory variables

During slipping operations, data was collected on several variables hypothesized to have the potential to affect slipping behaviour. These were: net hauling rate for the final 7/8^th^ of the net, amount of fish being slipped and the dimensions of the discharge channel.

Video footage from the bridge camera was used to calculate the net hauling rate, by observation of the speed at which the net entered the Triplex net roller. For this, the final 7/8^th^ of the net length (during which slipping typically took place) was marked at 10m intervals along the float-line by high visibility plastic tags, and their time of arrival at the Triplex noted. Due to the nature of purse seine fishing and lack of accurate monitoring equipment, it was not possible to directly measure the amount of fish being slipped. Amount of slipped catch was therefore estimated (in tonnes) by an experienced crew member informed by prior experience, as well as estimation of school size prior to shooting and movement of the fishing gear in response to the captured school. The width, depth and cross-sectional area of the discharge opening during slipping was modelled [33]. For this, depth was recorded every 5 seconds from up to seven RBR depth loggers attached at regular intervals along the bunt end. Additionally, the distance of the bunt float to the vessel was recorded using a laser range finder (Nikon Laser 550A S).

### Ethics statement

Permission to develop new slipping methodologies and allocation of associated scientific fishing quota was granted by the Directorate of Fisheries (Fiskeridirektoratet), the national authority regarding fisheries management for Norway. Note that slipping is a legal practice providing regulations [22] are adhered to. No other specific permissions were required for the work, and the work did not involve endangered or protected species. Additionally, no approval from local animal welfare authorities was required to conduct the work. This is because the collection of video images during routine fishing operations and during slipping has no additional impact on the welfare of the organisms.

### Data analysis

The collected data was summarised in three different datasets; an individual level behaviour dataset comprised of TBF observations and two collective level behaviour datasets.

#### The datasets

The TBF dataset (“Dataset A”, S1 Dataset) expressed number of tail beats per second, taking into account the number of video frames the fish were observed over and the known frame rate of the camera. “Dataset B” (S2 Dataset) expressed the proportion each collective behaviour (Table 1) contributed to the overall time budget per slipping event, excluding times when the fish did not escape. Single fish and small group escape events were highly transient and were therefore assumed to have one second durations. “Dataset C” (S3 Dataset) expressed the most dominant behaviour in consecutive 10 second time bins during different slipping events. For this, the proportion that each behaviour (Table 1) contributed to each 10 second bin was calculated. Instances of disagreement behaviours were divided equally between orderly and disorderly behaviours. The behaviour with the largest proportional contribution in each bin was then selected as the most dominant behaviour. In some time bins, orderly and disorderly escape behaviour contributed the same proportion. In these cases, a precautionary approach was taken and disorderly escape behaviour was chosen as the most dominant. For cases when “not in view” behaviour contributed the highest proportion and other behaviours were present, the next most common behaviour was taken as the dominant behaviour.

For all datasets, data exploration followed the protocol described in [45]. Slipping events for which no fish were caught or in which no behaviour was recorded were excluded from data analysis. Single fish and small group escapes were rare events, so were grouped together into a new behavioural category termed “small” for the purposes of data analysis. Net hauling rate (hereafter “rate”) was collinear with vessel and species, while discharge channel dimensions (“depth”, “width” and “area”) were highly collinear with one another. Therefore, these terms were never included in the same model together. Following the removal of missing covariate values in Dataset B, it was found that 90% of mackerel data came from one vessel alone. To avoid further collinearity issues, “species” and “vessel” were not included in the same model for this dataset.

#### Modelling the data

All statistical analysis was undertaken with R version 3.4.2 [46]. The datasets were hierarchically structured, in that multiple observations of slipping events were conducted upon different vessels and on different trips. Mixed effects modelling was therefore applied to Dataset A and Dataset C, with random covariate effects of “slipping event” nested within “trip”. Insufficient levels of “vessel” (number: 2) meant that the correlation structure could not be accurately determined [47], and vessel was therefore included as a fixed effect. Mixed modelling fitting procedures followed those described by [48]. Where appropriate, model assumptions were checked visually using residual plots, examining fitted values, covariates included and not included in the model and normality. Significance of the terms in the most parsimonious models were determined by likelihood ratio testing (LRT).

In order to determine factors driving TBF in mackerel and herring during slipping, a linear mixed model (LMM) was developed based on Dataset A, considering the covariates of”vessel” (categorical with two levels), as well as “escape type” (categorical with two levels, either large group or not), “amount” (slipped amount, continuous), “species” (categorical with two levels, either mackerel or herring), “observation” (categorical with two levels, either during or after escape) and their interactions. Backward selection was then applied, dropping the most insignificant term (p > 0.05) at each step to determine the most parsimonious model. However, the “species” term was never dropped to ensure that a model was fitted for both species. LMMs were fitted with the lme function from the nlme library of R [49].

Dataset B was used to determine important factors driving the observed slipping behaviour. The dataset was modelled using Dirichlet regression, a multivariate extension of beta regression. Dirichlet regression is appropriate for modelling response variables that represent proportions of a whole, corresponding to the behavioural composition of each slipping event. Further, Dirichlet regression can model compositional data that shows skewness and heteroscedasticity [50]. We employed an information-theoretic approach [51], in which 17 models were developed using combinations of the variables; “area” (cross sectional area of the discharge channel, continuous), “depth” (depth of the discharge channel, continuous), “width” (width of the discharge channel, continuous), “rate”, “amount” (square root transformed to reduce the influence of obvious outliers), “species” and their interactions, corresponding to specific hypotheses (Table 2) and considering the findings of the data exploration. Candidate models were ranked according to AIC corrected for small sample size (AICc, n = 26) and their normalized Akaike weights (AICw). The most parsimonious model was the one with the smallest AICc score and the largest AICw. Dirichlet regression was performed using DirichReg function of the DirichletReg package in R [52].

**Table 2:** Candidate Dirichlet regression models. Candidate models and associated hypotheses to explain the behavioural composition of fish whilst being slipped from purse seines.

Model	Covariates	Hypothesis
M1	Area + Amount + Area:Amount	Escape behaviour is determined by the amount of fish being released and the space available for them to escape the net
M2	Area + Species + Species:Area	Escape behaviour is determined by the species of fish being released and the available space for them to escape the net
M3	Depth + Species + Depth:Species	Escape behaviour is determined by the species of fish being released and the depth of the discharge opening
M4	Width + Species + Width:Species	Escape behaviour is determined by the species of fish being released and the width of the discharge opening
M5	Area + Vessel + Area:Vessel	Escape behaviour is determined by the vessel releasing and the available space for fish to escape the net
M6	Depth + Vessel + Depth:Vessel	Escape behaviour is determined by the vessel releasing and the depth of the discharge opening
M7	Width + Vessel + Width:Vessel	Escape behaviour is determined by the vessel releasing and the width of the discharge opening
M8	Rate + Area + Rate:Area	Escape behaviour is determined by how fast the net is hauled and the space available for escaping the net
M9	Amount + Species + Amount:Species	Escape behaviour is determined by the species and amount of fish being released
M10	Amount + Vessel + Amount:Vessel	Escape behaviour is determined by the vessel doing the slipping and how much they are releasing
M11	Amount + Rate + Amount:Rate	Escape behaviour is determined by the amount of fish being released and how fast the net is hauled
M12	Area	Escape behaviour is determined by the area of the discharge channel alone
M13	Vessel	Escape behaviour is determined by how the vessel conducts slipping
M14	Species	Escape behaviour is determined by the species of fish being released
M15	Amount	Escape behaviour is determined by the amount of fish exiting the net
M16	Rate	Escape behaviour is determined by how fast the net is hauled
M17	Null	None of the covariates affect how fish exit the purse seine

Dataset C was used to model the probability of fish escaping the purse seine over the duration of a slipping event, employing a generalized linear mixed model (GLMM) with a logit link function. A second GLMM was also developed, to model the probability of fish exiting in a “disorderly” manner (Table 1). For these models, a Bernoulli error structure was chosen, as the response variable (either escape/no escape or disorderly escape/other escapes) represented either successes or failures. Time bins were converted to a proportion of the total slipping time, to allow comparison between slipping events of different durations. Backwards selection based on dropping the least significant term was applied on the covariates of “vessel” and the interaction between “elapsed time” (proportion of the total slipping time, binomial) and “species” to arrive at the most parsimonious model. GLMMs were fitted using the glmer function of the lme4 library of R [53]. A pseudo R^2^ for the marginal (variance explained by the fixed effects) and conditional effects (variance explained by the fixed and random effects together) for mixed models was calculated using the equation of [54].

## Results

Observations of behaviour were collected from 39 slipping events across 8 different trips (Table 3). Of these events, 4 (10%) contained no usable behavioural data (Fig 1), either because the vessel failed to encircle the target school and the net was empty, or because no behavioural footage was recorded on the cameras. The discharge cameras were deployed on all events, while circumstances onboard meant that the drop camera was deployed on only 27 events (70% of all observed casts). Of the usable footage, the majority of observations (88%, n = 31) represented complete slipping events rather than partial slips. For both vessels combined, mean slipped amount was 158t (range: 1–1200t), while mean (± SD) width, depth and area of the discharge channels was 11 ± 2m, 7 ± 3m and 50 ± 25m^2^, respectively. Observations from individual slipping events consisted wholly of either mackerel or herring; mixed species catches were not encountered.

**Table 3:** Slipping events. Number of observed slipping events of mackerel and herring from purse seines.

Target species	Vessel	Trip no.	Date	No. of observed slipping events
Mackerel	Vessel A	1	September 2015	6
		2	October 2016	6
	Vessel B	1	June 2015	5
Herring	Vessel A	1	November 2015	1
		2	June 2016	6
	Vessel B	1	February 2015	5
		2	June 2016	4
		3	November 2016	6

### Collective behaviour observations

For slipping events in which collective behaviour was recorded (n = 35), the discharge channel was not in view for a mean of 57% (SD ±23%) of the total observed time. Disagreements between the behaviour recorded on either discharge cameras were rare, occurring on only 9 slips and totalling a mean of 1% (±1%) of the observed time. Return events occurred on 57% of slips; on average there was 3.6 (±3.1) return events per slip.

**Fig 4:**
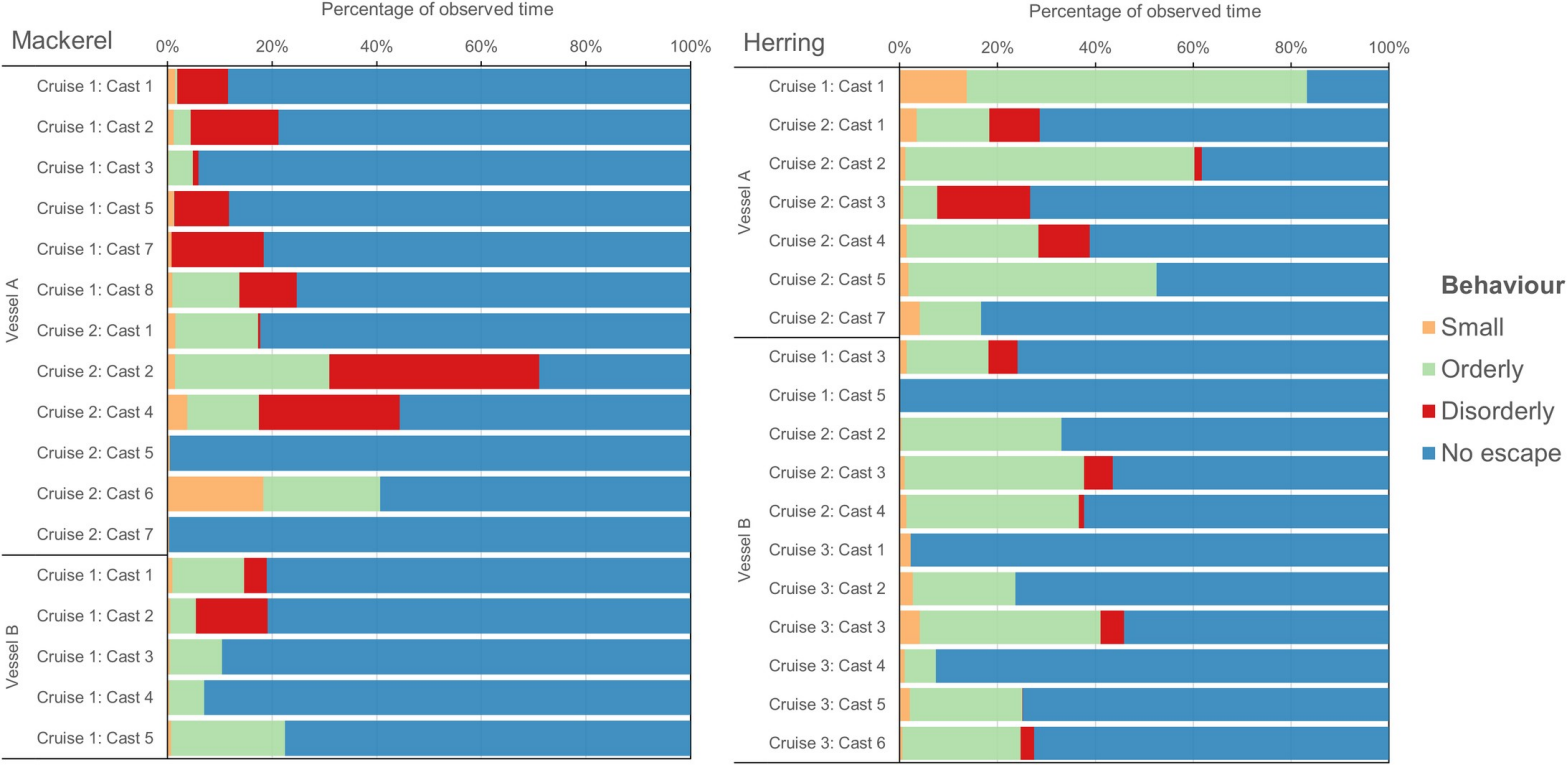
Behavioural time budget. The behavioural time budget of mackerel and herring whilst escaping from purse seines, from different slipping events (casts) from two different vessels.

Collective slipping behaviour varied considerably between slipping events (Fig 4). Of times when the discharge channel was in view, the majority of the slipping behaviour was comprised of “no escape” (mean ± SD per cast: 73 ± 20%). Of times when fish did escape the purse seine, the majority (84 ± 27%) escaped the net in either orderly (59 ± 35%) or disorderly (24 ± 31%) large groups; fish escaping individually or in small groups were relatively rare (15 ± 27%). Qualitative observations from the “drop camera” indicated that fish tended to swim with a downwards pitch after exiting the purse seine.

The composition of slipping behaviour differed between species and vessel. Mackerel showed a higher tendency for disorderly escapes than herring (39 ± 35% to 11 ± 19%, respectively), while orderly escapes tended to dominate slipping behaviour for herring more than mackerel (71 ± 31% to 47 ± 35%, respectively). Vessel A produced a higher proportion of disorderly escapes than Vessel B (37 ± 34% to 10 ± 19%, respectively), and a lower proportion of orderly escapes (49 ± 33% to 73 ± 33%, respectively).

### Development of collective behaviour over time

**Fig 5:**
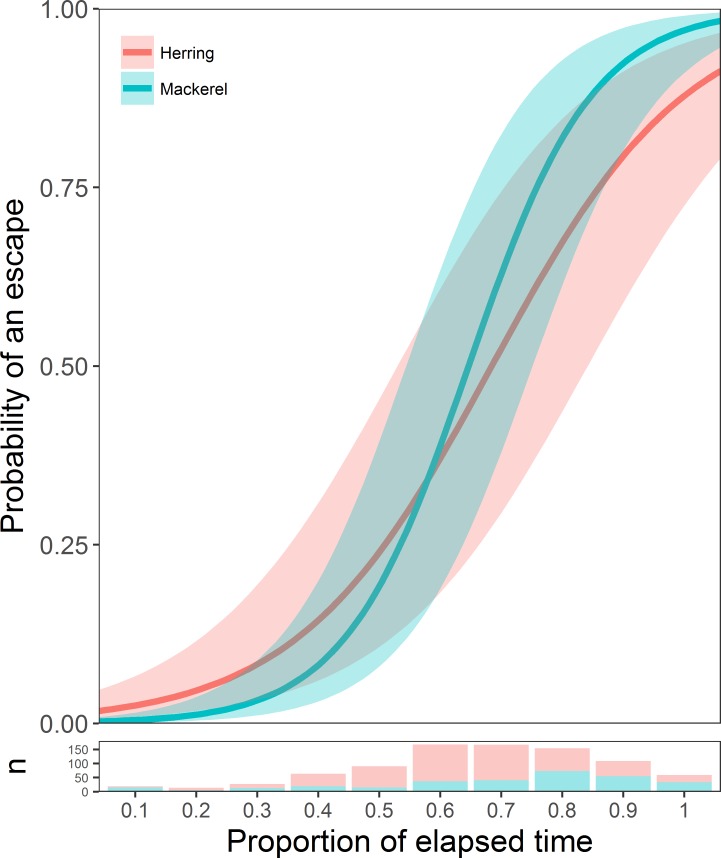
Probability of escape as a function of time. Estimated probability (with 95% confidence intervals) of a purse seine escape of any kind over time, for mackerel and herring. The lower panel shows the number of observed escape events per tenth-part of elapsed time. The dataset includes observations from both Vessel A and Vessel B.

The most parsimonious GLMM describing the probability of escaping the purse seine contained the fixed effect of an interaction between elapsed time and species (LRT, df = 1, LRT = 25.29, p = < 0.001). The variance explained by the model’s fixed effects was relatively low (marginal pseudo R^2^ = 0.37), while the fixed and random effects together accounted for substantially more (conditional pseudo R^2^ = 0.72), indicating large differences in the timings of escapes between different slipping events. For both species, the probability of an escape after the discharge opening was opened was initially low but increased over time (Fig 5). For mackerel and herring, there was a predicted 50% probability of an escape at 60% and 63% respectively of the elapsed time after the discharge channel was opened (Fig 5).

**Fig 6:**
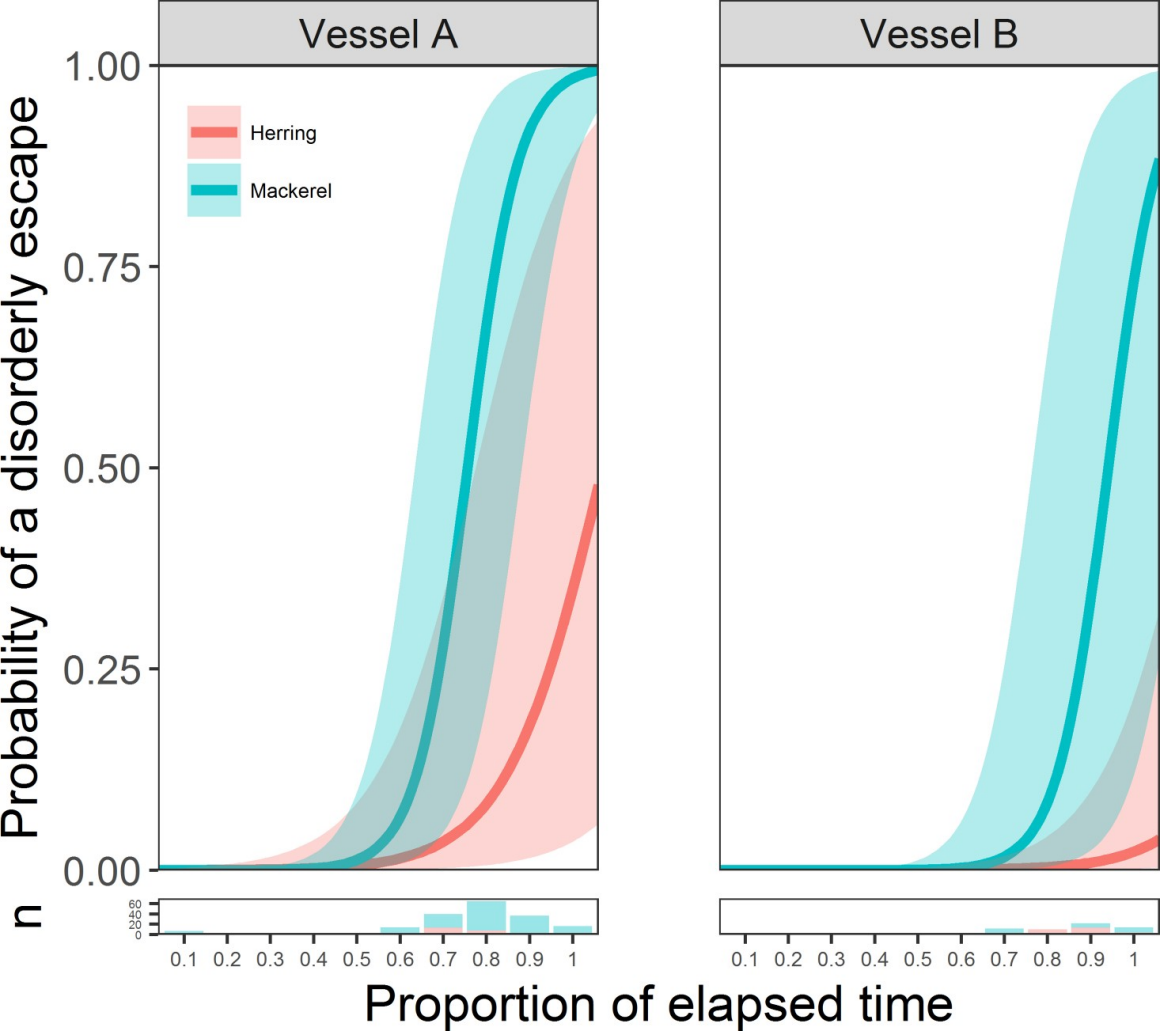
Probability of disorderly escape as a function of time. Estimated probability (with 95% confidence intervals) of a disorderly purse seine exit over time for mackerel and herring from Vessel A and Vessel B. The lower panels show the number of observed escape events per tenth-part of elapsed time.

The best adequate GLMM to explain the probability of a disorderly escape contained the fixed effects of vessel and an interaction between elapsed time and species (vessel LRT, df = 1, LRT = 4.05, p = < 0.05, elapsed time:species interaction LRT, df = 1, LRT = 18.31, p = < 0.001). The conditional and marginal pseudo R^2^ (0.89 and 0.47 respectively) indicated that a considerable proportion of the model variance was contained within the random effects, suggesting there were large differences in the timing of disorderly escapes between different slipping events. For both species from both vessels, the probability of disorderly escapes increased with time, although the probability of disorder was always higher for mackerel (Fig 6). There were also clear differences between vessels, with estimated probabilities suggesting that, for a given time, disorderly behaviour developed sooner and was more likely to occur in both species when released from Vessel A (Fig 6).

Examination of the distribution of escape behaviour over time (S1 Fig) showed that large group escapes would occur mainly in large “blocks”, indicating that once escaping began it would generally continue (with occasional small interruptions) until the net was empty. However, on occasion, escapes manifested as “bursts”, with longer periods of no escapes between “blocks”.

### Drivers of collective behaviour

Of our 17 models fitted by Dirichlet regression, models containing the discharge channel dimensions or hauling rate covariates tended to be substantially less well supported than those containing amount, species or vessel (Table 4). Although there was considerable support for the top three models (ΔAICc < 2, Table 4), M10 was selected as the most parsimonious as determined by the lowest AICc and highest AICw values.

**Table 4:** Dirichlet model ranking. Ranking of candidate Dirichlet regression models to explain the behavioural composition of fish whilst slipping from purse seines.

Model	Covariates	df	Log-likelihood	AICc	ΔAICc	Weight
M10	Amount + Vessel + Amount:Vessel	12	56.680	-65.361	0.000	0.380
M15	Amount	6	40.650	-64.880	0.481	0.299
M17	Null	3	35.668	-64.245	1.116	0.218
M14	Species	6	38.612	-60.803	4.557	0.039
M12	Area	6	38.163	-59.905	5.455	0.025
M13	Vessel	6	37.947	-59.473	5.887	0.020
M7	Width + Vessel + Width:Vessel	12	53.470	-58.939	6.421	0.015
M16	Rate	6	35.931	-55.440	9.921	0.003
M9	Amount + Species + Amount:Species	12	51.251	-54.501	10.859	0.002
M5	Area + Vessel + Area:Vessel	12	46.053	-44.107	21.254	<0.001
M1	Area + Amount + Area:Amount	12	45.067	-42.134	23.227	<0.001
M4	Width + Species + Width:Species	12	44.023	-40.045	25.316	<0.001
M11	Amount + Rate + Amount:Rate	12	42.869	-37.738	27.623	<0.001
M8	Rate + Area + Rate:Area	12	42.413	-36.825	28.536	<0.001
M6	Depth + Vessel + Depth:Vessel	12	42.177	-36.354	29.007	<0.001
M2	Area + Species + Species:Area	12	41.206	-34.412	30.949	<0.001
M3	Depth + Species + Depth:Species	12	41.049	-34.099	31.262	<0.001

The selected model (M10) contained amount, vessel and an interaction between these terms (Table 5) and predicted the behavioural composition significantly better than the null model (M17) (LRT, df = 9, LRT = 42.025, p = < 0.001). The interaction term had a highly significant effect (LRT, df = 3, LRT = 18.529, p = < 0.001) on the composition of slipping behaviour.

**Table 5:** Dirichlet regression results of the most parsimonious model. Parameters of the best selected model to explain the behavioural composition (comprised of small, orderly and disorderly behaviours) of fish slipped from purse seines, fitted by Dirichlet regression.

Behaviour	Variable	Estimate	SE	Z	*p*
Disorderly	(intercept)	-1.455	0.455	-3.195	<0.01
	Amount	0.110	0.033	3.352	<0.001
	Vessel (Vessel B)	-0.986	0.758	-1.302	0.193
	Amount : Vessel (Vessel B)	0.190	0.077	2.472	0.013
Orderly	(intercept)	-0.626	0.549	-1.139	0.255
	Amount	0.069	0.038	1.799	0.072
	Vessel (Vessel B)	-0.272	0.853	-0.318	0.750
	Amount : Vessel (Vessel B)	0.307	0.082	3.765	<0.001
Small	(intercept)	-0.938	0.498	-1.883	0.059
	Amount	0.034	0.034	0.993	0.321
	Vessel (Vessel B)	-1.510	1.035	-1.459	0.145
	Amount : Vessel (Vessel B)	0.271	0.095	2.835	<0.01

**Fig 7:**
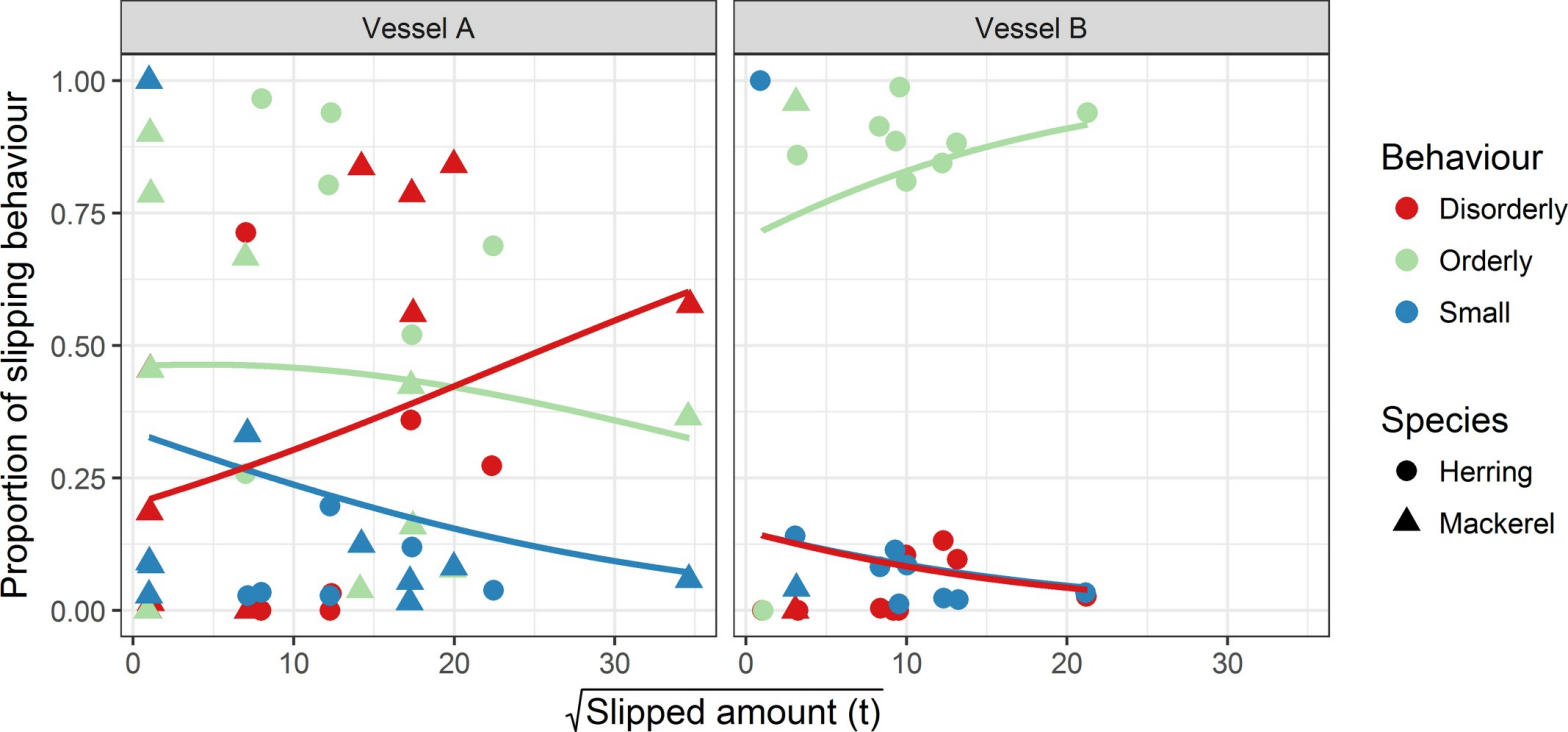
Behavioural composition as a function of slipped amount. The relationship between slipped amount and the composition of slipping behaviour (comprised of the behavioural units of disorderly, orderly and small) for herring and mackerel released by two different purse seine vessels. For Vessel B, note that the regression lines for “Disorderly” and “Small” overlap.

Predictions from the model (Fig 7) indicated that for Vessel A, an increasing amount of slipped fish resulted in an increasing proportion of disorderly escapes, with a simultaneous reduction in the proportion of fish escaping in an orderly way or in small groups/individually. For Vessel B, the situation was reversed; slightly decreasing proportions of disorderly and small group/individual escapes with increased slipped amount were predicted, while orderly escapes were predicted to increase (Fig 7). Dropping the extreme slipped amount datapoint at ~35^2 tonnes for Vessel A from the dataset did not substantially alter coefficient estimates or overall inferences drawn from the model.

### Individual level behaviour

**Fig 8:**
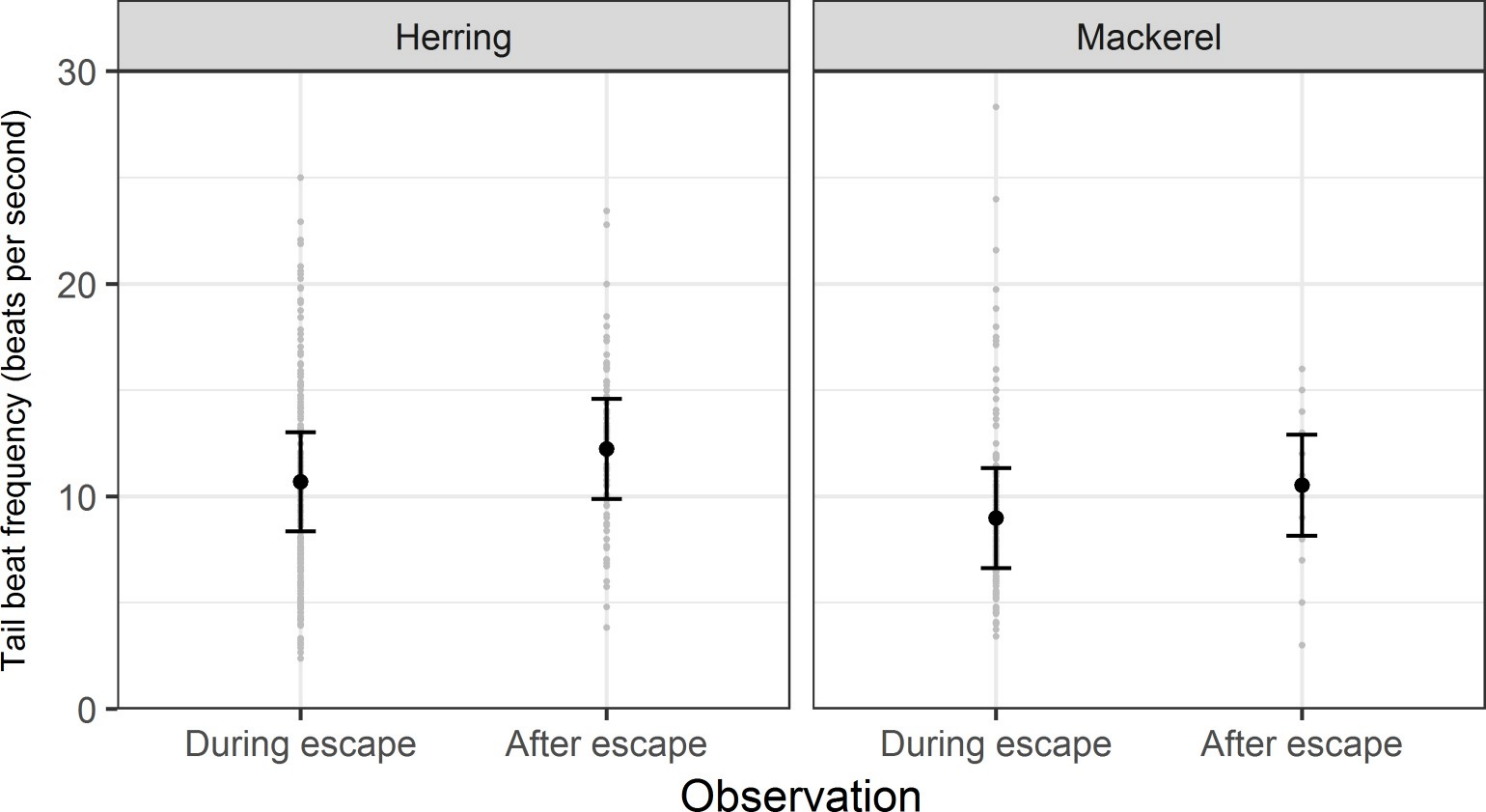
Tail beat frequency during and after escape. Model predicted mean (with 95% confidence intervals) tail beat frequency of mackerel and herring during and after slipping from purse seines.

The selected model to explain tail beat frequency (TBF) during escape for mackerel and herring contained “observation” and species as covariates. The covariates of “escape type” (large group or not), “slipped amount” and “vessel” were not statistically useful predictors of TBF. The observation position (either during or after slipping) significantly predicted TBF (LRT, df = 1, LRT = 34.9, p = < 0.001), whilst species did not (LRT, df = 1, LRT = 1.36, p = > 0.05). The conditional pseudo R^2^ was 0.39; marginal R^2^ was 0.08, suggesting large differences in TBF between different slipping events. Model predicted mean values indicated that both species tended to increase their TBF after escape (by 15% for herring and by 17% for mackerel, Fig 8). Furthermore, TBF was considerably more variable during escape compared with after escape, particularly for mackerel.

## Discussion

Efforts to maximise post slipping survival through better catch control earlier in the capture process (such as those described by [55] and [56]), may be negated if the slipping process itself further compromises the welfare of released catches. As such, it is important to understand how slipping methodology affects welfare. The results of this study describe the behaviour of mackerel and herring during slipping from purse seines, and highlight factors affecting the observed behaviour. To our knowledge, these observations are the first to describe fish behaviour whilst slipping from purse seines in the field.

Previous work on slipping has focused mainly on either small [20], mid-sized [17,57] or large-scale experiments [18,19,21] to estimate post slipping mortality and characterize physiological responses or has addressed specific topics such as the extent of slipping in particular fisheries [13]. [58] demonstrated that the method of slipping can significantly impact subsequent survival. However, none of these studies have examined behaviour or welfare implications in the field during the slipping process itself, although [59] showed impaired behaviour in response to predation following laboratory simulated slipping and [56] demonstrated that intra- and post-crowding behavioural responses could be used as a welfare indicator in mackerel.

The majority of slipped fish escaped the purse seine in a way which was likely to have minor welfare impacts (orderly escapes), as part of a large group in one self-contained period of time. These escapes typically took place towards the end of the slipping event. Fish escaping in such a way maintained schooling structure and therefore also presumably schooling function [60], with a low risk of individual physical injury by avoiding contact with conspecifics and the fishing gear. However, a large proportion of fish escaped purse seines in a way in which was likely to impact negatively upon their future survival potential (disorderly escapes), as schooling structure broke down and contact with conspecifics and gear occurred. Mackerel and herring are particularly vulnerable to mortality following physical injury [17,56,61–64]. Furthermore, a loss of predator avoidance, increased cost of locomotion, reduced information transfer rates and impaired foraging benefits can be expected when schooling structure fails [60].

For most of the time that the discharge opening was open, fish failed to escape and the probability of an escape increased over time. This result may be explained by two hypotheses; either the fish had difficulties finding the discharge channel or they were able to find it but were unwilling to use it. We propose that the second explanation is more likely, as we observed times when schools were extremely close to the discharge channel but did not escape; showing a reluctance to cross the threshold presented by the discharge opening/bunt end (see example in Fig 3). This explanation is further supported by the propensity for “return” events in which escaped fish turned and re-entered the net. Taken together with the fact that most fish escaped in large groups, these results indicate that individual fish were reluctant to leave the net because to do so would mean abandoning the anti-predator advantages of being in a school [60], in a situation they likely perceive as highly threatening. This suggests the current slipping method is dependent upon fish being effectively “forced” out of the net by the reduction in available volume as the net is hauled and the concurrent increase in negative stimuli (increasing noise, visual stimuli of netting approaching, unavoidable close proximity to conspecifics, etc.) as that process takes place. This contention is supported by the increase in probability of disorderly escapes for both species with increasing time. It is likely that as the net is hauled further after the formation of the discharge opening, the volume inside the purse seine reaches levels in which normal schooling behaviour is restricted and negatively perceived stimuli reach a high intensity, resulting in disorderly behaviour. Therefore to minimize negative effects on welfare, efforts should be made to develop ways to encourage fish to escape earlier in the slipping process, perhaps by altering the available visual stimuli or contrast of the discharge opening and/or the bunt end of the purse seine [65].

If the welfare of slipped purse seine catches is to be ensured it is important to identify the causes of such behaviour. Species was a key determinant of disorderly escape behaviour; for mackerel, there was a higher tendency towards disorderly escapes compared to herring. This observation matches previous work demonstrating a higher mortality rate for mackerel [19] than herring [21] in large scale slipping trials and species-specific differences in the response to purse seine capture [66,67]. The reasons for these behavioural differences are likely linked to differences in sensory modalities and the ability to respond to stimuli associated with purse seine capture. For instance, mackerel are likely less sensitive to sound [66] and have stronger swimming ability than herring [68]. However, the key sense in the maintenance of schooling is vision [69], and as such, differences in the ability to detect visual stimuli or differing responses and reaction thresholds to visual stimuli likely account for the difference. Although [70] noted schooling mackerel turned or swam erratically in response to visual stimuli, no authors have examined differences in the school level responses to visual stimuli between the two species. Some species-specific regulation of slipping practices is incorporated in current legislation [22] but may need to be expanded to fully account for this effect. In light of the fact that slipping behaviour appears to be species specific, future studies should work to quantify behaviour in other species targeted by purse seine, such as capelin (*Mallotus villosus*) and sardine.

The amount of fish released had a clear effect on slipping behaviour. Increases in the proportion of schooling structure breakdown (disorderly escapes) were seen with increasing amounts of slipped fish for one vessel, while the opposite trend was seen for the other. As the volume of the net decreases during hauling, the available space for schooling is reduced, increasing school density and especially so for larger catches [55]. It is therefore likely that differences in school density account to some degree for why slipped amount affects slipping behaviour. Schooling is integral to the life history strategies of mackerel and herring, allowing the rapid transfer of information between individuals and resulting in complex and coordinated collective level responses to stimuli [71]. This is achieved by adherence by schoolmates to simple behavioural rules of far field attraction, near field repulsion and alignment [72]. Importantly, the speed and effectiveness of information transfer is dependent on school density [73] and an increase in coordinated responses typically accompanies increases in schooling density [74]. Larger (and consequently, denser) catches should therefore theoretically be more coordinated upon slipping, meaning less disorderly and more orderly escapes. We observed this effect on only one of the vessels (Vessel B). It would therefore seem that for the other vessel, increases in density caused the individual behavioural rules that define coordinated schooling to break down for a larger proportion of the catch. The reason for this difference between vessels is not immediately clear, as both operated with nets of similar sizes and therefore similar changes in school density with hauling can be expected. However, as the majority of observations from Vessel B were comprised of herring, a species effect cannot be fully discounted here and indeed, the Dirichlet model containing the “species” covariate alone was relatively well supported. Taken together, these results suggest for some conditions, the slipping method examined here is increasingly unsuitable as the amount of slipped fish increases, and alternative methods for such situations may need to be developed.

In all of the mixed modelling, the variance explained by the conditional effects was considerably larger than fixed effects alone, indicative of a high degree of variation in the manifestation of escape behaviour between different slipping events. Diversity in the behavioural response of different fish schools to purse seine capture has been observed previously [66,67,75,76]. In these cases, differences may have arisen due to differences in behavioural stimuli between different capture events, such as light levels between night and day [66,75] or the particular hydrographic conditions which may have altered vessel generated stimuli such as noise [76]. Other explanations include school specific factors such as difference in initial activity level [66] or position within the purse seine upon encirclement [67]. It is likely such factors also play an important role in determining the expression of slipping behaviour as well, and may therefore help to explain why we observed such large variations in behaviour between different slipping events. Factors such as biological condition (which varies considerably between seasons for mackerel and herring) or behavioural state of the school prior to encirclement may also influence subsequent slipping behaviour. For example, a school previously under attack from a predator can be expected to differ in response to capture than a school simply migrating. Of particular importance to slipping behaviour however may be wind and current conditions, which dictate the extent that side thruster propellers are used by the vessel. Thrusters likely introduce extreme auditory stimuli at close range to the fish, as well as deforming the shape of the net and should be monitored in future studies examining slipping behaviour. This said, the diversity of factors potentially affecting behaviour highlights the difficulty in minimizing welfare impacts on slipped catches in a consistent manner.

The dimensions of the discharge opening were likely of an adequate size to release at least small schools without interfering with behaviour substantially. However, we observed instances of disorderly behaviour in even the smallest slipped amounts (≤1 tonne). Conversely, the relatively small size of the discharge opening in comparison to the larger catch sizes we observed (up to 1200 tonnes) would make it highly improbable that such a large mass of fish could be released without at least some effect upon their behaviour. These facts together with the result that models containing discharge opening dimensions were not well supported, suggests that slipping behaviour was primarily determined inside the bunt end prior to escape. However, from our observations, we were unable to determine if this was the case due to the positioning of the cameras. Such knowledge would further inform science-based regulation of slipping practices and may be gathered by linking behavioural observations of schooling behaviour inside the purse seine to behaviour at the point of slipping.

Although the effects of species and vessel could not be fully resolved by Dirchelet regression, the GLMM results indicate a clear effect of vessel in disorderly escape probability. Fish slipped from Vessel A had a higher probability of welfare compromising behaviour than fish from Vessel B for any given time during slipping. The reasons for this are likely related to vessel specific behavioural stimuli not encompassed by the covariates we monitored, as well as the particular handling style of the gear by the fishers. It should be noted that purse seine nets in the water are dynamic, adopting different shapes depending, in part, on how they are handled [2,55]. Therefore, net structure may cause localized areas of high schooling density, resulting in school structure breakdown. It is worthwhile to highlight that the vessel which minimized disorderly escape behaviour was the coastal vessel. Norwegian coastal purse seiners (including Vessel B, J. Saltskår, *pers*. *comm*.) have a tradition of live transfer and storage of catches to holding pens [61], to enable them to take catches in excess of their hold capacity as well as allowing some optimisation of when the catch is sold. This experience could result in more welfare friendly slipping practices. Whatever the explanation, it is encouraging that certain vessels have the capacity to release fish in a welfare friendly manner probably due primarily to their handling of the gear, and future work should focus on identifying these handling parameters.

Examination of tail beat frequency (TBF) allowed us to examine behaviour on the level of individual fish. For both species, TBF increased following escape from the purse seine. A positive correlation between TBF and swimming speed has been reported for many species [40,77,78], including mackerel [79] and herring [80]. It is therefore reasonable to assume, based on TBF, that fish increased their swimming speed following escape from the purse seine. Comparison of swimming speed between mackerel and herring based on our data is however invalid, due to differences in stride length (the distance covered per tail beat) between species and individual level-effects (eg. differences in sizes of individual fish [38]). Drawing inferences on the welfare implications of this increase in speed is also difficult; while the animal may move away from the capture related stressors inside the net more rapidly when swimming faster, it could also be forced to respond beyond its normal physiological capacity [81] thereby making such a response maladaptive.

Increases in swimming speed in small pelagic species have been noted previously in response to stressors [35,82], as the fish attempt to avoid threats. As such, one might expect that swimming speed would be reduced after escape, as there would presumably be a reduction in negatively perceived stimuli once outside the net. However, this was not the case. It could be that the dimensions of the discharge opening restricted free-swimming movements for fish in large groups, but in that case, differences in TBF between fish escaping individually or in small groups would be expected and this effect was not observed. More likely is that the restrictive volume available for swimming inside the net meant that individual fish could not swim in a straight line and were forced to swim in either circular milling patterns (as suggested by [67]) or with pronounced and numerous alterations in direction. Fish not swimming in straight lines can be expected to have lower speeds [83]. It is probable that this restriction in speed was evident when fish first escaped the net but was removed allowing the fish to accelerate by the time they passed the “drop camera” a few seconds later. This suggestion is supported by the observation that schools were always polarised and coordinated after escaping; that is, their swimming path was straighter. [59] examined behaviour in sardines (*Sardina pilchardus*) and found a reduction in swimming speed after simulated purse seine slipping. The difference between this and our results can be explained by the timing and nature of the observations; [59] examined swimming speed up to 3 days post-slipping in the laboratory while we examined speed immediately post-escape in the field.

For the majority of time, the discharge channel was out of the field of view of the cameras, preventing any quantification of slipping behaviour during these occasions. Although this will have reduced our overall observed time, it is unlikely to have influenced our overall conclusions regarding the behavioural composition and timings of slipping events. The discharge cameras were attached to the discharge opening itself, and as the opening moved around in the water in response to the current and waves, so the cameras moved, sometimes encompassing the discharge channel in their field of view and at other times not. Therefore, our observations can be thought of as random snapshots of escape behaviour throughout the slipping event, with no systematic biases. Recent advances in underwater video technology [84] may, however, allow future observations to be conducted at distance from the discharge channel, avoiding the need to attach cameras to moving gear and thereby increasing the amount of observed time.

There are potentially additional explanatory variables that influence slipping behaviour which we did not include in our analysis. The duration of the fishing operation up to the point of slipping often varies due to operational reasons onboard and may therefore have an important effect on determining behaviour upon escape due to differences in the duration of exposure to capture related stressors. Furthermore, as herring and mackerel are ectothermic, their potential for behavioural activity is determined to some degree by temperature. Our observations likely encompass a range of temperatures due to the large temporal and spatial extent of our study, but we did not record this parameter. Likewise, schooling is chiefly mediated visually [69] meaning that differences in lighting conditions (including night versus day) between casts may have further influenced schooling behaviour. The majority of our dataset consisted of complete slipping events, meaning that for these cases slipped amount was a fair proxy of crowding density prior to slipping. However, it may have been informative to relate behavioural composition to crowding density for the few casts in our dataset which represented partial slips. Accounting for variables such as these may have helped to further explain the variability in the composition of the collective slipping behaviour we observed between different casts and vessels.

In conclusion, the results show that the slipping method examined primarily releases fish in a manner that is likely to have minor welfare impacts. However, the method can also induce behaviours that may be indicative of compromised welfare and subsequent survival. The results also indicate that the probability that welfare compromising behaviour will be expressed is situation specific, and depends on the particular slipping event, the species being released, the particular vessel releasing the fish, how long the discharge opening has been open and the amount of fish being released. In order to improve slipping methodologies, further investigation is needed to highlight additional factors affecting behaviour which may further explain the observed variability between events, and to definitively link slipping behaviour to subsequent mortality.

## Supporting information

S1 Video “No escape” video sequence. An example of the “No escape” behavioural unit, recorded by the horizontally orientated discharge camera.
(MP4)

S2 Video “Single fish” video sequence. Examples of the “Single fish” behavioural unit, recorded by the vertically orientated discharge camera.
(MP4)

S3 Video “Small group” video sequence. An example of the “Small group” behavioural unit, recorded by the horizontally orientated discharge camera.
(MP4)

S4 Video “Orderly” video sequence. Examples of the “Orderly” behavioural unit, with persepctives recorded by the both the horizontally orientated and the vertically orientated discharge cameras.
(MP4)

S5 Video “Disorderly” video sequence. Examples of the “Disorderly” behavioural unit, recorded by the horizontally orientated discharge camera.
(MP4)

S6 Video “Return” video sequence. Examples of the “Return” behavioural unit, with persectives recorded by the both the vertically orientated and the horizontally orientated discharge cameras (equipped with red light).
(MP4)

S7 Video “Drop camera” video sequence. Examples of the perspective given by the drop camera.
(MP4)

S1 Dataset “Dataset A”. Dataset containing observations of tail beat frequency during and after escape from purse seines.
(CSV)

S2 Dataset “Dataset B”. Dataset of behavioural time budgets per slipping event.
(CSV)

S3 Dataset “Dataset C”. Dataset of the most dominant slipping behaviour per 10 second interval of slipping.
(CSV)

S1 Fig The most dominant slipping behaviour patterns over time for mackerel and herring from two different vessels. Vertical bar represent 10 second bins. A: Mackerel from Vessel A; B: Mackerel from Vessel B; C: Herring from Vessel A; D: Mackerel from Vessel B.
(TIF)

## References

[1] Bell JD, Watson RA, Ye Y. Global fishing capacity and fishing effort from 1950 to 2012. Fish Fish. 2017; 18(3): 489–505.

[2] Ben-Yami M. Purse seining manual. Oxford; Cambridge, MA, USA: Published by arrangement with the Food and Agriculture Organization of the United Nations (FAO) by Fishing News Books; 1994.

[3] Suuronen P, Chopin F, Glass C, Løkkeborg S, Matsushita Y, Queirolo D, et al Low impact and fuel efficient fishing—Looking beyond the horizon. Fish Res. 2012; 119: 135–146.

[4] Schau EM, Ellingsen H, Endal A, Aanondsen SA. Energy consumption in the Norwegian fisheries. J Clean Prod. 2009; 17: 325–334.

[5] Tyedmers P. Fisheries and energy use In: Cleveland C, editor. Encyclopedia of Energy. New York: Elsevier; 2004 pp. 683–693.

[6] Basurko OC, Gabiña G, Uriondo Z. Energy performance of fishing vessels and potential savings. J Clean Prod. 2013; 54: 30–40.

[7] Breen M, Isaksen B, Ona E, Pedersen AO, Pedersen G, Saltskår J, et al A review of possible mitigation measures for reducing mortality caused by slipping from purse-seine fisheries. ICES CM 2012/C:12 Available from:http://www.ices.dk/sites/pub/CM%20Doccuments/CM-2012/C/C1212.pdf

[8] Chopin FS, Arimoto T. The condition of fish escaping from fishing gears—a review. Fish Res. 1995; 21(3–4): 315–327.

[9] Broadhurst MK, Suuronen P, Hulme A. Estimating collateral mortality from towed fishing gear. Fish Fish. 2006; 7(3): 180–218.

[10] Mesnil B. When discards survive: accounting for survival of discards in fisheries assessments. Aquat. Living Resour. 1996; 9: 209–215.

[11] Crowder LB, & Murawski SA. Fisheries bycatch: implications for management. Fisheries 1998; 23(6): 8–17.

[12] Breen M, Cook R. Inclusion of discard and escape mortality estimates in stock assessment models and its likely impact on fisheries management. ICES CM. 2002; 27: 1–15.

[13] Stratoudakis Y, & Marçalo A. Sardine slipping during purse-seining off northern Portugal. ICES J. Mar. Sci. 2002; 59(6): 1256–1262.

[14] Borges L, Van Keeken OA, Van Helmond AT, Couperus B, Dickey-Collas M. What do pelagic freezer-trawlers discard? ICES J. Mar. Sci. 2008; 65(4): 605–611.

[15] Marçalo A, Breen M, Tenningen M, Onandia I, Arregi L, Gonçalves JM. Mitigating Slipping-Related Mortality from Purse Seine Fisheries for Small Pelagic Fish: Case Studies from European Atlantic Waters In: Uhlmann SS, Ulrich C, Kennelly SJ editors. The European Landing Obligation. Cham: Springer; 2019 pp 297–318.

[16] Davis MW. Key principles for understanding fish bycatch discard mortality. Can. J. Fish. Aquat. Sci. 2002; 59(11): 1834–1843.

[17] Lockwood SJ, Pawson MG, Eaton DR. The effects of crowding on mackerel (*Scomber scombrus* L.)—physical condition and mortality. Fish Res. 1983; 2(2): 129–147.

[18] Mitchell RW, Blight SJ, Gaughan DJ, Wright IW. Does the mortality of released *Sardinops sagax* increase if rolled over the headline of a purse seine net? Fish Res. 2002; 57(3): 279–285.

[19] Huse I, Vold A. Mortality of mackerel (*Scomber scombrus* L.) after pursing and slipping from a purse seine. Fish Res. 2010; 106(1): 54–59.

[20] Marçalo A, Marques TA, Araújo J, Pousao-Ferreira P, Erzini K, Stratoudakis Y. Fishing simulation experiments for predicting the effects of purse-seine capture on sardine (*Sardina pilchardus*). ICES J. Mar. Sci. 2010; 67(2): 334–344.

[21] Tenningen M, Vold A, Olsen RE. The response of herring to high crowding densities in purse-seines: survival and stress reaction. ICES J. Mar. Sci. 2012; 69(8): 1523–1531.

[22] Fiskeridirektoratet. Section §48a in Regulations Relating to Sea-water Fisheries. 2014 Available from: https://lovdata.no/dokument/SF/forskrift/200412221878/KAPITTEL_12#KAPITTEL_12

[23] Diggles BK, Cooke SJ, Rose JD, Sawynok W. Ecology and welfare of aquatic animals in wild capture fisheries. Rev. Fish Biol. Fish. 2011; 21(4): 739–765.

[24] Torgersen T, Bracke MB, Kristiansen TS. Reply to Diggles et al (2011): Ecology and welfare of aquatic animals in wild capture fisheries. Rev. Fish Biol. Fish. 2011; 21(4): 767–769.

[25] Huntingford FA, Adams C, Braithwaite VA, Kadri S, Pottinger TG, Sandøe P, et al Current issues in fish welfare. J Fish Biol. 2006; 68(2): 332–372.

[26] Veldhuizen LJL, Berentsen PBM, de Boer IJM, van de Vis JW, Bokkers EAM. Fish welfare in capture fisheries: A review of injuries and mortality. Fish Res. 2018; 204: 41–48.

[27] Browman HI, Cooke SJ, Cowx IG, Derbyshire SW, Kasumyan A, Key B, et al Welfare of aquatic animals: where things are, where they are going, and what it means for research, aquaculture, recreational angling, and commercial fishing. ICES J. Mar. Sci. 2018; 10.1093/icesjms/fsx225

[28] Digre H, Hansen UJ, Erikson U. Effect of trawling with traditional and ‘T90’ trawl codends on fish size and on different quality parameters of cod *Gadus morhua* and haddock *Melanogrammus aeglefinus*. Fish Sci. 2010; 76: 549–559.

[29] Schreck CB, Olla BL, Davis MW. Behavioral responses to stress. In: Iwama GK, Pickering AD, Sumpter JP, Schreck CB, editors. Fish stress health in aquaculture; 1997 pp. 145–170.

[30] Dawkins MS. Using behaviour to assess animal welfare. Anim Welf. 2004; 13 (1): 3–7.

[31] Breder CM. On the survival value of fish schools. Zoologica. 1967; 52(2): 25–40.

[32] Fernö A. Advances in understanding of basic behaviour: consequences for fish capture studies. ICES MSS. 1993; 196: 5–11.

[33] Vold A, Anders N, Breen M, Saltskår J, Totland B, Øvredal JT. Beste praksis for slipping fra not. Rapport fra Havforskningsinstituttet 2017; 6 Available from: https://www.hi.no/filarkiv/2017/03/beste_praksis_slipping_fra_notvold_et_al.pdf/nb-no

[34] Prado J, Dremiere PY. Fisherman’s Workbook. Oxford: Fishing News Books; 1990.

[35] Martin P, Bateson PPG, Bateson P. Measuring behaviour: an introductory guide. 2^nd^ ed Cambridge: Cambridge University Press; 1993.

[36] Rieucau G, Holmin AJ, Castillo JC, Couzin ID, Handegard NO. School level structural and dynamic adjustments to risk promote information transfer and collective evasion in herring. Anim Beh. 2016; 117:489 69–78.

[37] Misund OA, Aglen A. Swimming behaviour of fish schools in the North Sea during acoustic surveying and pelagic trawl sampling. ICES J. Mar. Sci. 1992; 49(3): 325–334.

[38] Herbert NA, Steffensen JF. Hypoxia increases the behavioural activity of schooling herring: a response to physiological stress or respiratory distress? Mar Bio. 2006; 149(5): 1217–1225.

[39] Videler JJ, Wardle CS. Fish swimming stride by stride: speed limits and endurance. Rev. Fish Biol. Fish. 1991; 1(1): 23–40.

[40] Steinhausen MF, Steffensen JF, Andersen NG. Tail beat frequency as a predictor of swimming speed and oxygen consumption of saithe (Pollachius virens) and whiting (Merlangius merlangus) during forced swimming. Mar Bio; 2005; 148(1): 197.

[41] Leonard JBK, Norieka JF, Kynard B, McCormick SD. Metabolic rates in an anadromous clupeid, the American shad (*Alosa sapidissima*). J Comp Physiol B. 1999; 169(4–5): 287–295.

[42] Ohlberger J, Staaks G, Hölker F. Estimating the active metabolic rate (AMR) in fish based on tail beat frequency (TBF) and body mass. J Exp Zool A Ecol Genet Physiol. 2007; 307(5): 296–300. 10.1002/jez.384 17366622

[43] Lowe C. Metabolic rates of juvenile scalloped hammerhead sharks (*Sphyrna lewini*). Mar Biol. 2001; 139(3): 447–453.

[44] Schneider CA, Rasband WS, Eliceiri KW. NIH Image to ImageJ: 25 years of image analysis. Nat Methods. 2012; 9(7): 671–5. 22930834 10.1038/nmeth.2089 PMC5554542

[45] Zuur AF, Ieno EN & Elphick CS. A protocol for data exploration to avoid common statistical problems. Methods Ecol Evol. 2010; 1: 3–14.

[46] R Core Team. R: A language and environment for statistical computing [Internet]. Vienna, Austria: R Foundation for Statistical Computing; 2017 Available: https://www.R-project.org/

[47] Crawley MJ. Statistical Computing: An Introduction to Data Analysis Using S-PLUS. Chichester: John Wiley & Sons; 2002.

[48] Zuur A, Ieno EN, Walker NJ, Saveliev AA, Smith GM. Mixed effects models and extensions in ecology. New York: Springer-Verlag; 2009.

[49] Pinheiro J, Bates D, DebRoy S, Sarkar D, R Core Team. nlme: Linear and Nonlinear Mixed Effects Models [Internet]. R package version 3.1; 2017 Available: https://CRAN.R-project.org/package=nlme.

[50] Maier MJ. DirichletReg: Dirichlet Regression in R [Internet]. R package version 0.6; 2015 Available: http://dirichletreg.r-forge.r-project.org/

[51] Burnham KP and Anderson DR. Model Selection and Multimodel Inference: A Practical Information-Theoretic Approach, 2nd ed New York: Springer; 2002.

[52] Maier MJ. DirichletReg: Dirichlet regression for compositional data in R. Vienna: Research Report Series; 2014: Report 125.

[53] Bates D, Maechler M, Bolker B, Walker S. Fitting Linear Mixed-Effects Models Using lme4. J Stat Softw. 2015; 67(1): 1–48.

[54] Nakagawa S, Schielzeth H. A general and simple method for obtaining R^2^ from generalized linear mixed-effects models. Methods Ecol Evol. 2013; 4(2): 133–142.

[55] Tenningen M, Peña H, Macaulay GJ. Estimates of net volume available for fish shoals during commercial mackerel (*Scomber scombrus*) purse seining. Fish Res. 2015; 161: 244–251.

[56] Handegard NO, Tenningen M, Howarth K, Anders N, Rieucau G, Breen M. Effects on schooling function in mackerel of sub-lethal capture related stressors: Crowding and hypoxia. PloS One. 2017; 12(12): e0190259 10.1371/journal.pone.0190259 29284035 PMC5746257

[57] Pawson MG, Lockwood SJ. Mortality of mackerel following physical stress, and its probable cause. ICES MSS. 1980; 177: 439–443.

[58] Marçalo A, Guerreiro PM, Bentes L, Rangel M, Monteiro P, Oliveira F, et al Effects of different slipping methods on the mortality of sardine, *Sardina pilchardus*, after purse-seine capture off the Portuguese Southern coast (Algarve). PloS One. 2018; 13(5), e0195433 10.1371/journal.pone.0195433 29851955 PMC5978792

[59] Marçalo A, Araújo J, Pousão‐Ferreira P, Pierce GJ, Stratoudakis Y, Erzini K. Behavioural responses of sardines *Sardina pilchardus* to simulated purse‐seine capture and slipping. J. Fish Biol. 2013; 83(3): 480–500. 10.1111/jfb.12184 23991869

[60] Pitcher TJ, Parrish JK. Functions of shoaling behavior in teleosts In: Pitcher TJ, editor. Behaviour of Teleost Fishes. London: Chapman & Hall; 1993 pp. 363–439.

[61] Misund OA, Beltestad AK. Survival of herring after simulated net bursts and conventional storage in net pens. Fish Res. 1995; 22(3–4): 293–297.

[62] Suuronen P, Erickson DL, Orrensalo A. Mortality of herring escaping from pelagic trawl codends. Fish Res. 1996; 25(3–4): 305–321.

[63] Misund OA, Beltestad AK. Survival of mackerel and saithe that escape through sorting grids in purse seines. Fish Res. 2000; 48(1): 31–41.

[64] Olsen RE, Oppedal F, Tenningen M, Vold A. Physiological response and mortality caused by scale loss in Atlantic herring. Fish Res. 2012; 129: 21–27.

[65] Glass CW, Wardle CS, Gosden SJ, Racey DN. Studies on the use of visual stimuli to control fish escape from codends. I. Laboratory studies on the effect of a black tunnel on mesh penetration. Fish Res. 1995; 23(1–2): 157–164.

[66] Misund OA. Avoidance behaviour of herring (*Clupea harengus*) and mackerel (*Scomber scombrus*) in purse seine capture situations. Fish Res. 1993; 16(2): 179–194.

[67] Tenningen M, Macaulay GJ, Rieucau G, Peña H, Korneliussen RJ. Behaviours of Atlantic herring and mackerel in a purse-seine net, observed using multibeam sonar. ICES J. Mar. Sci. 2016; 74(1): 359–368.

[68] He P. Swimming speeds of marine fish in relation to fishing gears. ICES MSS. 1993; 196: 183–189.

[69] Breder CM. Studies on social groupings in fishes. Bull. Am. Mus. Nat. Hist. 1959; 117: 393–482.

[70] Cui G, Wardle CS, Glass CW, Johnstone ADF, Mojsiewicz WR. Light level thresholds for visual reaction of mackerel, *Scomber scombrus* L., to coloured monofilament nylon gillnet materials. Fish Res. 1991; 10(3–4): 255–263.

[71] Handegard NO, Boswell KM, Ioannou CC, Leblanc SP, Tjøstheim DB, Couzin ID. The dynamics of coordinated group hunting and collective information transfer among schooling prey. Curr Biol. 2012; 22(13): 1213–1217. 10.1016/j.cub.2012.04.050 22683262

[72] Katz Y, Tunstrøm K, Ioannou CC, Huepe C, Couzin ID. Inferring the structure and dynamics of interactions in schooling fish. Proc. Natl. Acad. Sci. U.S.A. 2011; 108(46): 18720–18725. 10.1073/pnas.1107583108 21795604 PMC3219116

[73] Sumpter D, Buhl J, Biro D, Couzin I. Information transfer in moving animal groups. Theory Biosci. 2008; 127(2): 177–186. 10.1007/s12064-008-0040-1 18458976

[74] Buhl J, Sumpter DJ, Couzin ID, Hale JJ, Despland E, Miller ER, et al From disorder to order in marching locusts. Science. 2006; 312(5778): 1402–1406. 10.1126/science.1125142 16741126

[75] Misund OA. Sonar observations of schooling herring: school dimensions, swimming behaviour, and avoidance of vessel and purse seine. ICES MSS. 1990; 189: 135–146.

[76] Misund OA. Predictable swimming behaviour of schools in purse seine capture situations. Fish Res. 1992; 14(4): 319–328.

[77] Bainbridge R. The speed of swimming of fish as related to size and to the frequency and amplitude of the tail beat. J Exp Zool. 1958; 35(1): 109–133.

[78] Tu Z, Yuan X, Han J, Shi X, Huang Y, Johnson D. Aerobic swimming performance of juvenile *Schizothorax chongi* (Pisces, Cyprinidae) in the Yalong River, southwestern China. Hydrobiologia. 2011; 675(1): 119–127.

[79] Wardle CS, He P. Burst swimming speeds of mackerel, *Scomber scombrus* L. J. Fish Biol. 1988; 32(3): 471–478.

[80] Handegard NO, Pedersen G, Brix O. Estimating tail-beat frequency using split-beam echosounders. ICES J. Mar. Sci. 2009; 66(6): 1252–1258.

[81] Wood CM, Turner JD, Graham MS. Why do fish die after severe exercise? J. Fish Biol. 1983; 22(2): 189–201.

[82] Misund OA, Aglen A. Swimming behaviour of fish schools in the North Sea during acoustic surveying and pelagic trawl sampling. ICES J. Mar. Sci. 1992; 49(3): 325–334.

[83] He P, Wardle CS. Endurance at intermediate swimming speeds of Atlantic mackerel, *Scomber scombrus* L., herring, *Clupea harengus* L., and saithe, *Pollachius virens* L. J. Fish Biol. 1988; 33(2): 255–266.

[84] Risholm P, Thorstensen J, Thielemann JT, Kaspersen K, Tschudi J, Yates C, et al Real-time super-resolved 3D in turbid water using a fast range-gated CMOS camera. Appl Opt. 2018; 57(14): 3927–3937. 10.1364/AO.57.003927 29791362

