# Supplementary figures and images for: Behavioural and welfare implications of a new slipping methodology for purse seine fisheries in Norwegian waters
Source: PLoS One. 2019 Mar 11;14(3):e0213031. doi: 10.1371/journal.pone.0213031 (PMC6411124; doi:10.1371/journal.pone.0213031)

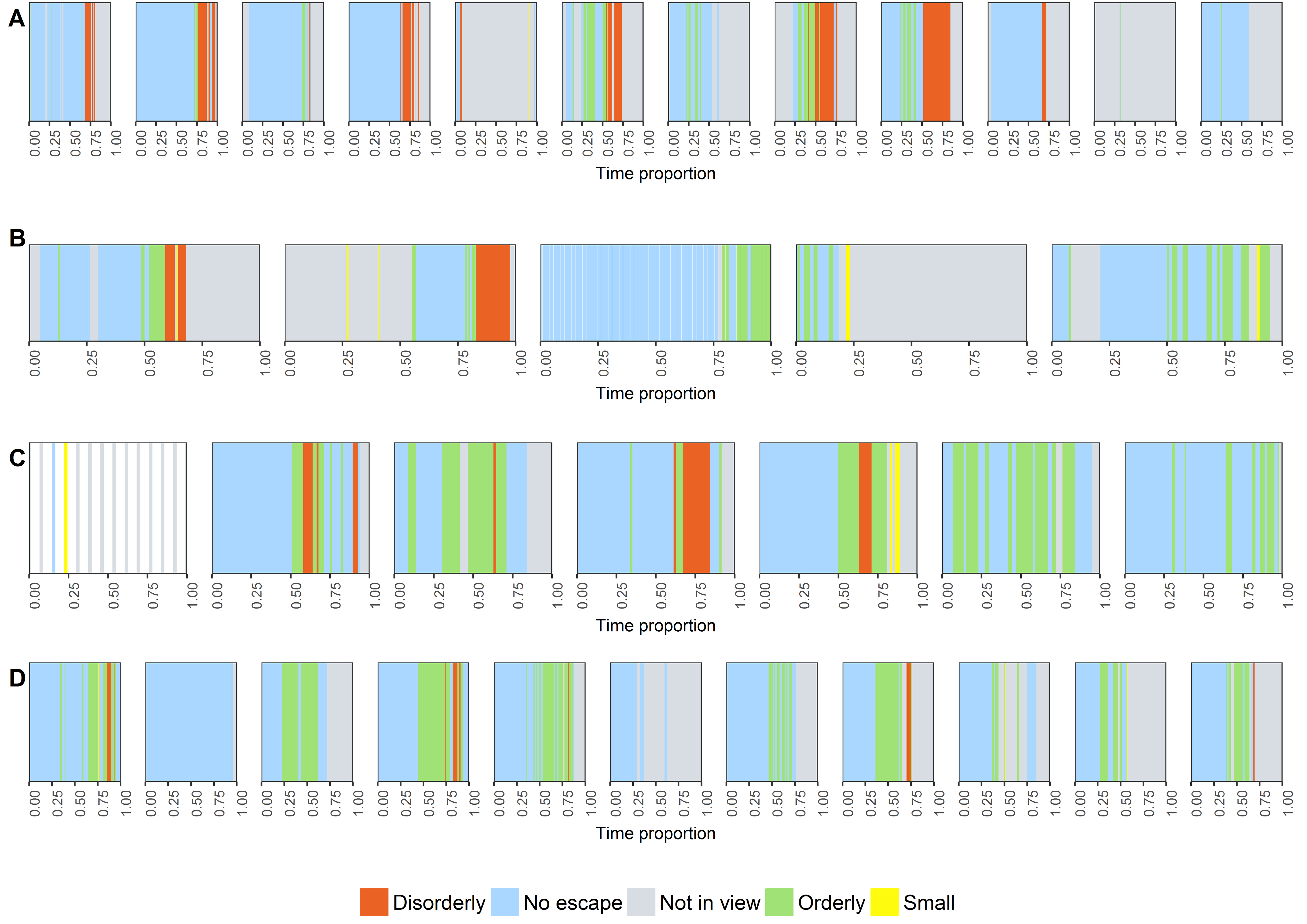

Supplement: S1 Fig — Vertical bar represent 10 second bins. A: Mackerel from Vessel A; B: Mackerel from Vessel B; C: Herring from Vessel A; D: Mackerel from Vessel B. (TIF) [file pone.0213031.s011.tif]
